# Validation of a semi-automatic threshold-based approach for right ventricular endocardial border detection for volumetry

**DOI:** 10.1186/1532-429X-18-S1-P165

**Published:** 2016-01-27

**Authors:** Tamadhir F Gazzaz, Paweena Chungsomprasong, Shi-Joon Yoo, Mike Seed, Lars Grosse-Wortmann

**Affiliations:** The Hospital for Sick Children, Toronto, ON Canada

## Background

Cardiac magnetic resonance imaging (CMR) is the gold standard for evaluation of right ventricular (RV) volumes, function, and mass. The conventional postprocessing approach is based on ‘manual' delineation of the endocardium/blood border. The use of a threshold-based algorithm offers the theoretical advantage of a more inclusive definition of the RV myocardium, within a shorter postprocessing time.

## Methods

**Figure 1:**
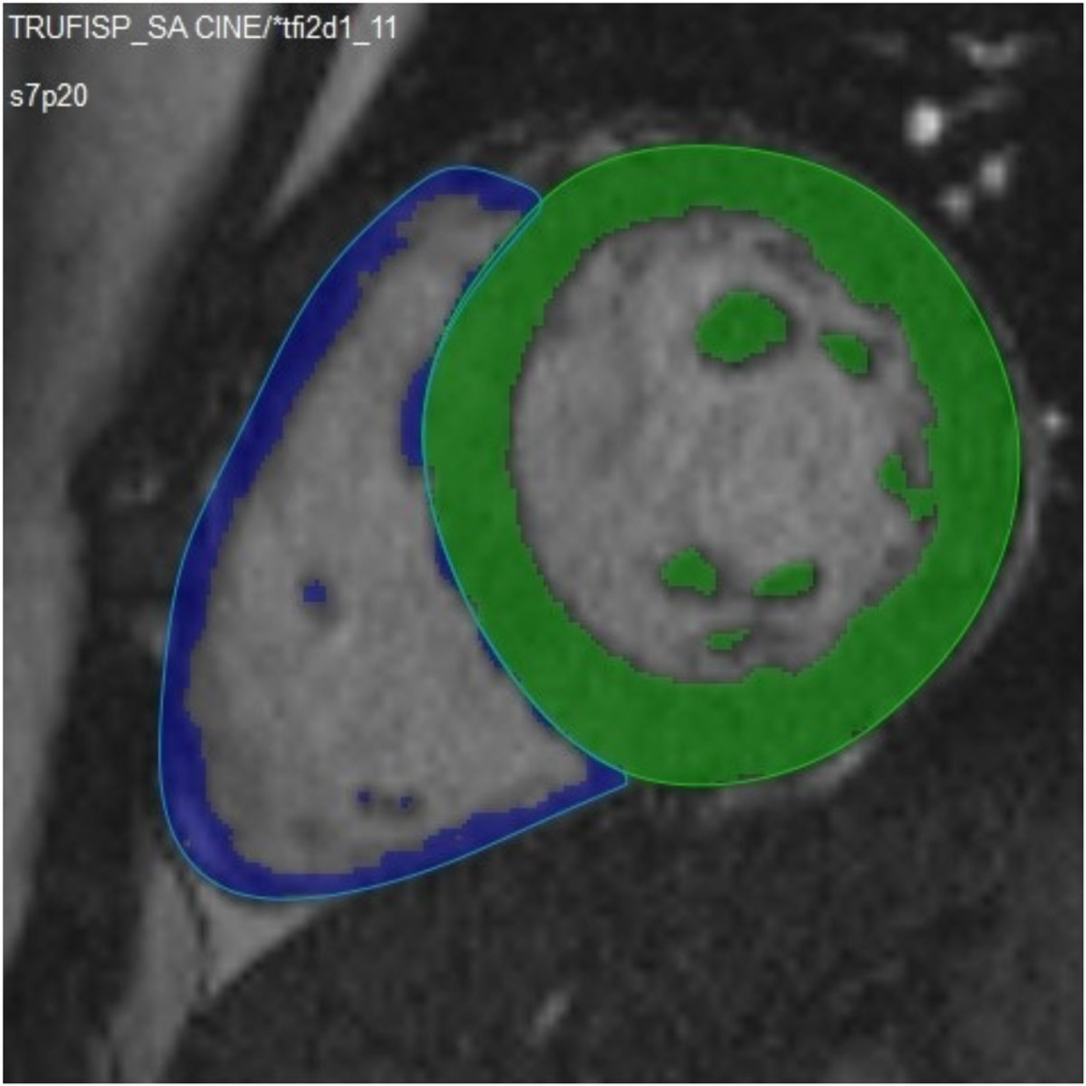
**Semi-automatic endocardial contour detection using a threshold algorithm**. The epicardial contours are manually drawn.

60 children (mean age 13.9 ± 2.7 years) including 20 with structurally normal hearts and 40 after Tetralogy of Fallot (TOF) repair who underwent clinical CMR studies were included. The second group included 20 selected patients with good image quality and 20 unselected consecutive TOF studies (differences in performance of the volumetry approaches for patient subgroups are clinically important but reporting this information is beyond the constraints of an abstract). A cine short axis stack was acquired for ventricular volumetry. The RV endocardial border was identified by three methods: A) manual contouring with exclusion of RV trabeculations from the myocardium, B) including RV trabeculations, and C) semi-automatic thresholding (SAT) (Fig.1).

## Results

The SAT method took less time than either variant of the conventional method (12.6 ± 2.2 min vs.17.5 ± 2.4 min by method A and 19.5 ± 2.9 min by method B,respectively, p < 0.001 for both). RV end-diastolic volume (EDV) measured by SAT was lower than that by conventional approaches (bias -11.4 ml/m^2^ in comparison with A and -6.9 ml/m^2^ with B). There was good agreement of SAT RV SV with both conventional methods (bias -0.4 ml/m^2^ and -1.8 ml/m^2^,respectively). Expectedly, RV mass by SAT was higher than by conventional methods (bias 13.2 gm/m^2^ and 8.8 gm/m^2^,respectively). The correlation between RV SV and MPA forward flow was moderate and comparable between all three methods (r^2^ 0.53-0.57). The bias between SAT derived SV and MPA forward flow was between that of methods A and B (bias 5.7 ml/m^2^, 5.2 ml/m^2^, and 6.9 ml/m^2^ for SAT, A, and B,respectively). SAT RV SV as well as EDV had higher interobserver variability compared to both conventional methods (bias -9.0 ml/m² vs. 4.2 ml/m^2^ and 0.83 ml/m^2^,respectively for SV and 5.4 ml/m² vs.1.43 ml/m^2^ and 2.81 ml/m^2^, respectively for EDV). Interobserver reproducibility for RV mass was superior by SAT as compared to the conventional method A and B (bias -4.0 gm/m² vs.-0.35 gm/m² and -2.7 gm/m^2^,respectively).

## Conclusions

The semi-automated threshholding method for RV endocardial border detection is faster compared to the conventional methods. RV EDV by SAT are smaller and RV mass greater than by conventional approaches. Reproducibility for RV EDV was lower and that for RV mass was better by SAT. The accuracy compared to MPA flow measurements are comparable between all three approaches. SAT is an alternative postprocessing strategy with potential advantages for RV mass measurements. Operator training in a uniform contouring / threshholding approach remains a prerequisite for successful quantification of RV mass and volumes.

